# Reactive Thrombocytosis Associated with Acute Myocardial Infarction following STEMI with Percutaneous Coronary Intervention

**DOI:** 10.1155/2013/707438

**Published:** 2013-11-24

**Authors:** Nat Dumrongmongcolgul, Charoen Mankongpaisarnrung, Grerk Sutamtewagul, Nattamol Hosiriluck, Timothy Chen, Alexander Trujillo, Nicholas Dcunha, Kenneth Nugent, Leigh Ann Jenkins

**Affiliations:** Department of Internal Medicine, Texas Tech University Health Sciences Center, 3601 4th Street, Indiana Avenue, Lubbock, TX 79430, USA

## Abstract

The etiology of thrombocytosis can be classified into reactive and essential forms. The rate of thromboembolic events is higher in essential thrombocytosis, and these events include strokes, transient ischemic attacks, retinal artery or retinal vein occlusions, digital ischemia, and acute coronary syndrome. In a study of 732 medical and surgical patients with thrombocytosis, 88% had reactive thrombocytosis. Patients with reactive thrombocytosis do not require cytoreductive medications or antiplatelet treatment. We report a healthy 40-year-old man without any medical problems who developed a new episode of myocardial infarction associated with thrombocytosis after an episode of myocardial infarction followed by percutaneous coronary intervention. He had thrombocytosis, and his platelet function test did not reveal adequate inhibition. To treat his acute coronary syndrome, therapeutic enoxaparin was added, and clopidrogel was substituted with ticagrelor. We decided to start hydroxyurea to reduce platelets counts. Enoxaparin and hydroxyurea were discontinued when platelet count returned to baseline. JAK-2 and BCR/ABL mutations were negative. This case report highlights a clinical dilemma (reactive thrombocytosis), which is challenging in terms of management and pathophysiology.

## 1. Main Document

A 44-year-old man presented to the emergency department with shortness of breath and chest pain. His only cardiovascular risk factor was cigarette smoking. Physical examination revealed a blood pressure of 82/67 mmHg, heart rate of 132 beats per minutes, and body temperature of 97.2 F. His cardiovascular examination revealed normal first and second heart sounds, with no jugular venous distention, murmurs, rubs, or gallops. The remainder of his physical examination was unremarkable. Initial ECG was sinus tachycardia without ischemic ST-T changes. He developed an episode of witnessed cardiac arrest with ventricular fibrillation. Cardiopulmonary resuscitation (CPR) was performed for 40 minutes until the return of spontaneous circulation. The initial troponin T was 1.91 ng/mL (0.01–0.03) after the rhythm was reestablished, the electrocardiogram showed sinus tachycardia with ST-segment elevation in I, aVL, V4, V5, and V6. CK-MB was 181.4 ng/mL (0.1–0.49), and creatine kinase was 4248 units/L (77–308). Emergency coronary angiogram showed only total occlusion of left anterior descending artery. Left circumflex coronary artery and right coronary artery were normal. Percutaneous transluminal coronary angioplasty was performed with a pressure of 8 atmospheres for 14 seconds, and then a bare metal stent (Vision 2.75 × 18 mm (diameter × length)) was implanted in the mid third of the left anterior descending artery. Aspirin, carvedilol, simvastatin, and clopidogrel were initiated. After percutaneous coronary intervention (PCI), he was placed on therapeutic hypothermia protocol for 24 hours. The hospital course was uneventful. He was extubated and finally discharged home after 9 days without neurological consequences. His complete blood count upon discharge was white blood cell count 6.4 k/*μ*L, hemoglobin 12.1 g/dL, hematocrit 34.9%, and platelet count 349 k/*μ*L. 

**Figure 1:**
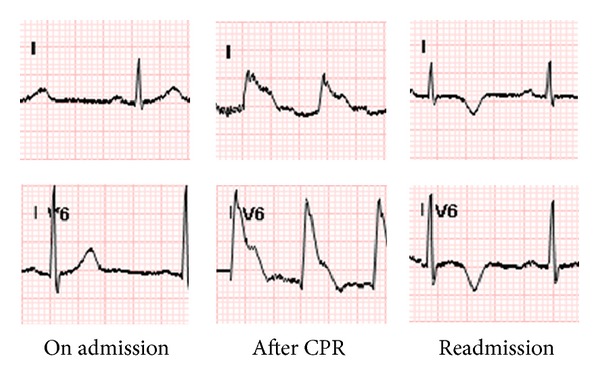
As shown, electrocardiogram on admission date was unremarkable; then, the ST segment elevation developed after cardiac arrest. New T wave inversion was found on readmission date.

He was readmitted three days later because of recurrent chest pain at rest, even though the patient had been compliant with medication. On admission, physical examination revealed a blood pressure of 86/46 mmHg, heart rate of 72 beat per minutes, body temperature of 97.6°F, and respiratory rate of 14/minutes. He was in acute distress; his lungs were clear to auscultation bilaterally. Hepatosplenomegaly was not appreciated. His platelet count was now 966 k/*μ*L, and the P2Y12 inhibition test (Verifynow) was 11%. (Therapeutic range ≥20%). His peripheral blood smear showed thrombocytosis without basophilia. Troponin T was 0.1 ng/mL, CK-MB was 10.3 ng/mL, and creatine kinase was 97 units/L. Ferritin level was 284 ng/mL. The electrocardiogram showed normal sinus rhythm with unchanged T wave inversion in V4, V5, and V6 but new T wave inversion in V2 and V3 (Figure 1). He was admitted to coronary intensive care unit and underwent cardiac catheterization, which demonstrated a patent stent. Therapeutic dose of enoxaparin was initiated, and clopidogrel was substituted with ticagrelor. The hematology service was subsequently consulted because of thrombocytosis, and hydroxyurea was initiated with the pending studies for JAK2 and BCR/ABL mutations. After three days of hospitalization, he was discharged home on hydroxyurea, ticagrelor, and therapeutic doses of enoxaparin. Two weeks later, his platelet count was 372 k/*μ*L, and JAK 2 and BCR/ABL mutations were negative. Enoxaparin and hydroxyurea were discontinued at that time. 

## 2. Discussion 

The etiology of thrombocytosis can be classified into reactive thrombocytosis and essential thrombocytosis. Reactive thrombocytosis is caused by elevated thrombopoietin level and other cytokines, such as interleukin-6. Essential thrombocytosis is a myeloproliferative disorder characterized by megakaryocytic hyperplasia in bone marrow and predisposes patients to vascular complications, such as bleeding or thrombosis [1]. The rate of thromboembolic events is higher in essential thrombocytosis, and these include strokes, transient ischemic attacks, retinal artery or retinal vein occlusions, digital ischemia, and acute coronary syndrome [2]. In a study of 732 medical and surgical patients with thrombocytosis, 88% had reactive thrombocytosis. The most common causes included tissue damage from surgery, infection, malignancy, and postsplenectomy status [3]. Of 187 patients diagnosed with essential thrombocytosis, 50% had at least one episode of thromboembolic events within nine years of diagnosis [4]. A mutation in the gene encoding Janus kinase 2 (JAK2) is present in 50% of patients with essential thrombocytosis; however, it can be found in healthy individuals [5, 6]. Thrombosis in postsplenectomized patients commonly occurs in portal system with platelet counts more than 650 k/*μ*L and has an incidence of approximately 5% [7]. Patients with reactive thrombocytosis do not usually require cytoreductive medication or antiplatelet treatment [8], but, in some circumstances, those with reactive thrombocytosis from postsplenectomy and iron deficiency anemia may require antiplatelet treatment given the potential risk of acute coronary syndrome, stroke, pulmonary embolism, or pulmonary hypertension [9–12]. 

Our patient developed reactive thrombocytosis after the episode of myocardial infarction followed by percutaneous coronary intervention. Upon discharge, platelet count returned to normal, but there was a dramatic increase to 996 k/*μ*L two days later (Table 1). Our literature search identified only one case report describing reactive thrombocytosis following the episode of myocardial infarction [13]. We think that this is the second reported case of reactive thrombocytosis following the myocardial infarction since other potential causes seem highly unlikely. However, thrombocytosis in our patient was possibly caused by injury from prolonged CPR. 

It is well documented that essential thrombocytosis is a predilection to myocardial infarction. Of interest, our patient might have developed another episode of acute coronary syndrome (ACS) from reactive thrombocytosis. He had a recurrent typical angina, new dynamic ST-T changes on V2 and V3, and a new rise of cardiac enzyme with the percentage from CK-MB to CK level of greater than 5%. Theoretically, CK-MB level usually returns to baseline in 4 days. With his acute presentation suggestive of acute coronary syndrome, stent thrombosis was a major concern. As a result, he underwent a second coronary angiography, which demonstrated a patent stent without significant thrombosis. Normal coronary angiograms can be found at 8–12% in patient with acute myocardial infarction [14]. The pathophysiology of acute coronary syndrome in this case is probably not from atherosclerosis or plaque rupture, and we hypothesize that the elevated platelet count from reactive thrombocytosis caused occlusion of a small artery in the myocardium or endocardium, resulting in a second myocardial infarction with normal coronary angiogram. Theoretically, a loading dose of 600 mg should produce a clopidrogrel peak plasma level in 2 hours and peak effect in 6 hours; 75 mg dose will produce 40% and 60% of inhibition of platelet activity in 3 and 7 days, respectively [15]. Resistance to clopidogrel is well described with higher incidence of major cardiac events within 30 days after PCI [11]. The inhibition of platelet activity in our patient was only 11% after a loading dose of 600 mg of clopidogrel and 75 mg daily for 10 days, suggesting that his platelets might not have been suppressed adequately by clopidogrel (drug failure), which led to a recurrent myocardial infarction. Another possibility is that he had primary resistance to clopidogrel and developed a second episode of myocardial infarction. In this scenario, the reactive thrombocytosis would be an incidental finding. We decided to substitute clopidogrel with a newer generation of antiplatelet medication, ticagrelor. To treat recurrence of ACS, therapeutic dose of enoxaparin was initiated to prevent further progression of secondary hemostasis. Hydroxyurea, aspirin, and ticagrelor were initiated. Enoxaparin and hydroxyurea were discontinued when his platelet count returned to his baseline. This case underscores the potentially underrecognized danger of reactive thrombocytosis. The role of antiplatelet agents or anticoagulant or even cytoreductive medication is not well established. More studies are needed for comprehensive evaluation of the pathophysiology, consequences, and management of reactive thrombocytosis.

## Figures and Tables

**Table 1:** Complete blood counts in relation to his hospital course.

Admission date	1	6	8	9	12	13	14	15	27	37
				DC	RA		HY		FU1	FU2
Platelet count k/*μ*L	266	162	212	249	966	851	830	857	372	237
White blood cell count k/*μ*L	6.4	6.4	4.8	6.4	10.1	7.0	6.9	2.6	4.5	4.8
Hemoglobin g/dL	15.4	11.6	12.1	11.8	12.1	11.6	11.7	12.1	13.1	13.2

DC: discharge date, RA: readmission date, HY: hydroxyurea start date, and FU: follow-up date.
